# A Practical Approach to Excessive Daytime Sleepiness: A Focused Review

**DOI:** 10.1155/2016/4215938

**Published:** 2016-05-12

**Authors:** Brian J. Murray

**Affiliations:** Division of Neurology, Department of Medicine, Sunnybrook Health Sciences Centre, University of Toronto, Toronto, ON, Canada M4N 3M5

## Abstract

Excessive daytime sleepiness (EDS) is a common problem that is important to recognize and address. Initial steps in management are generally straightforward and only the most advanced cases would require referral to a subspecialist. Of particular concern is that of driving safety. There is a broad differential diagnosis for conditions contributing to EDS but a few common conditions account for the majority of clinical presentations. Subjective self-reporting will often lead to identification of potential problems, but this is often unreliable. Traditional neurophysiologic tests can help in objectively quantifying symptoms but current tests are not always practical clinically and may have little validation in real world situations. There are many treatment options that should generally be able to sufficiently manage most patients presenting with hypersomnolence. This review provides a practical clinical approach to the problem based on current guidelines.

## 1. The Problem

Excessive daytime sleepiness (EDS) is a common clinical problem that is important to recognize and address. This review provides a practical approach to the diagnosis and management of excessive daytime sleepiness for the majority of adults based on current guidelines.

## 2. Review Strategy

A PubMed search for EDS in English language, with human subjects, and with available abstract produced 441 articles under the categories of practice guideline, review, and systematic review. These were reviewed by the author to identify relevant articles for this review format. References in this paper include only a few key summaries that have broad applicability as well as a few articles of particular interest. Only a few references were cited for this review format on the basis of the need for identifying some of the most useful sources for practicing clinicians and it is not meant to be exhaustive.

## 3. Clinical Approach

**Figure 1:**
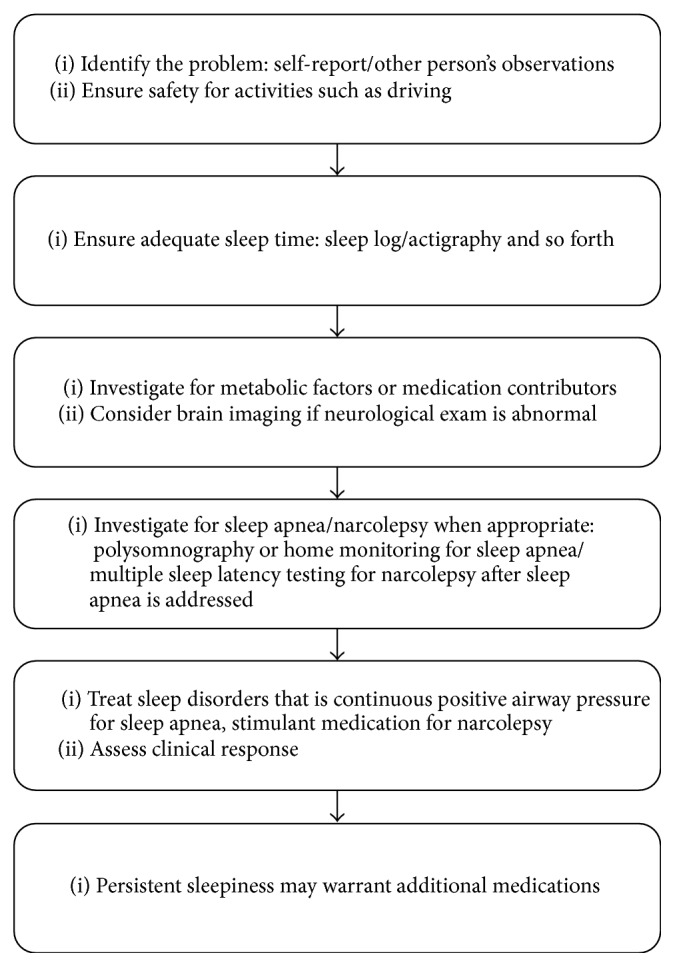
Clinical approach to excessive daytime sleepiness.

See Figure 1 for the general approach to clinical management. The first step is identifying EDS. The individual identifies EDS when it is creating a problem in their life such as falling asleep in inappropriate settings such as driving. The situation is even more concerning when colleagues, friends, or family have identified concerns, as subjective reporting may lead some individuals to deny the problem. Sleepiness can be hard to quantify as the subjective experience varies between individuals. In general, observable behaviors and objective measures are better used wherever possible. A physician may first identify sleepiness by seeing patients asleep in the waiting room.

Some patients may not identify sleepiness but will identify their problem as fatigue. Fatigue is vague word and may reflect sleepiness but also reflects attributes including pain, disability, and depression. There can be significant overlap in depression and EDS. Being tired, feeling run down, and having low energy may be seen in both. Clinically, tearfulness or hopelessness may identify more prominent mood symptoms.

There are many causes of EDS [1]. The first condition to identify is that of insufficient sleep. Most people need around 7–9 hours of sleep and many get less. If someone needs to wake up by an alarm clock, they are most likely getting insufficient sleep time. Tracking sleep times in the form of a sleep log can be helpful although it is important for the participant to record activities every day and not simply guess or summarize at the end of a longer period. Actigraphy, smartphones, and fitness devices can also be helpful tools to estimate sleep. Patients with shift work may develop profound sleepiness due to cumulative sleep deficit as well as being awake at times when there is a natural circadian tendency for profound sleepiness, for example, 03:00–05:00.

Metabolic problems can also contribute to sleepiness. Patients with anemia and hypothyroidism commonly report fatigue. A number of other metabolic disorders can produce sleepiness including hepatic and renal failure. Electrolyte abnormalities or systemic inflammation can also contribute. These conditions are generally identified on a basic medical examination and blood work.

Medications are another common contributor to sleepiness; psychoactive medications are particularly problematic. Many antidepressants, anxiolytics, pain medications, and antiepileptics impair alertness. Over-the-counter medications must also be considered; antihistamines are associated with somnolence, for example. Drugs and alcohol may also contribute to sleepiness.

After these factors are addressed, sleep apnea (SA) will be one of the most common presenting conditions. Increased weight, enlarged neck circumference, and airway abnormalities may be identified in addition to a report of snoring. The upper airway resistance syndrome (UARS) should also be carefully considered. Even if there is no significant oxygen desaturation, or scored hypopneas, sleep may be fragmented by mild obstructive events and sleep fragmentation and consequent daytime sleepiness results.

Less commonly, narcolepsy will be responsible for sleepiness. Narcolepsy occurs in about 1 in 2000 persons. While this is rare, these patients are more likely to present to a sleep specialist. EDS is the only symptom that must be present to establish a diagnosis. This can occur in the absence of cataplexy, sleep paralysis, and sleep onset hallucinations but if these symptoms are present, the condition is more likely.

Brain abnormalities can also impair alertness. Distortion of midline projecting systems by a mass lesion is notorious for producing somnolence. By disrupting midline projecting systems, head injuries can cause a reduction in orexin/hypocretin signalling similar to that seen in narcolepsy [2]. Many neurodegenerative conditions are associated with sleepiness. A careful neurologic examination may identify these conditions. Neuroimaging is suggested in most of these situations.

## 4. Measurement of Sleepiness

A simple tool that is commonly used in clinical practice is the Epworth Sleepiness Scale [3]. This is an 8-item scale that asks people to subjectively assess how likely they are to fall sleep in a variety of common situations. One study revealed an average score of 7.5 in a lineup of people waiting to renew their driver's licenses [4]. Many clinicians consider values above 10 as clinically significant.

Polysomnography is not useful to quantify sleepiness. However, polysomnography may be obtained for assessment of possible obstructive SA. Ambulatory monitoring devices may also suffice if there is a high likelihood of apnea. If a multiple sleep latency test is going to be pursued, this must follow a conventional polysomnogram.

There are published practice parameters on the use of neurophysiological alertness testing. The multiple sleep latency test (MSLT) is best used for the diagnosis of narcolepsy [5]. The MSLT should only be obtained after sleep disordered breathing is fully treated. In the MSLT, patients have 20 minutes to* fall asleep* and the test is repeated 4 or 5 times throughout the day separated by 2 hours. The test should be done off psychoactive medications after a normal sleep routine. A drug screen can help ensure there is no substance contributing to daytime sleepiness. Most clinicians consider a mean sleep latency under 10 minutes as clinically significant. Patients with narcolepsy have a mean latency less than 8 minutes. One advantage of the MSLT in particular is that the degree of sleepiness cannot be exaggerated by the patient. This may be helpful if a patient is seeking drugs of abuse, for example.

In contrast, the maintenance of wakefulness test (MWT) has better face validity for assessment of safety in a variety of situations. In this test, the patient gets 20–40 minutes to* stay awake*, and sessions are repeated every 2 hours for 4 or 5 attempts. There are advantages to the longer 40-minute testing in that it is more sensitive to detecting sleepiness. This test has been noted to be subject to motivational factors [6]. The study is not technically validated for driving safety, but many clinicians use the ability to remain awake on this test as a good marker of alertness.

Other tests that have been used in research settings include the psychomotor vigilance task, which is sensitive to sleep loss as well as circadian variation in alertness. The test is available on mobile devices, which could be used in clinical settings. Driving simulators have been of interest but none are widely available and will not address multiple potential safety concerns in different life settings. New tests are under development [7].

## 5. Treatment Options

Treatment options should start by ensuring that adequate sleep time is obtained. Strategic napping may also help [8]. Treatment of underlying sleep disorders such as SA with continuous positive airway pressure, for example, is essential.

A number of other simple measures may assist the subject with sleepiness. Standing up can improve alertness. Bright light is helpful, particularly with evening work. Caffeine is commonly used to enhance alertness [9]. Caffeine should be avoided beyond mid-afternoon if possible to minimize sleep disruption. Removing sedative substances is also important. Unnecessary medications that are sedating should be removed or switched to more alerting options if they are available. Some antidepressants, such as fluoxetine taken in the morning, for example, are alerting and this may represent a better choice over a sedating option such as amitriptyline. Sleeping pills have “hangover” effects that impair alertness the following day and should be avoided. Chronotherapeutics, or simply changing the timing of medication administration, may be helpful [10].

Once SA is treated, EDS is sometimes persistent. The UARS may be a contributing factor and should be addressed as best possible. As an adjunct to the treatment of SA, modafinil can be added [11]. Generally, a dose of 100 mg can be given in the morning and another at noon. This can be increased to a maximum of 400 mg daily. The medications should be administered at a time for when the patient is most sleepy. This medication is generally well tolerated, with only some reporting headache and extremely rare allergic skin reactions, with little abuse or cardiovascular complications.

Stimulant medications of other varieties can be used, particularly in conditions such as narcolepsy [12]. Longer acting agents such as slow release methylphenidate, for example, 20 mg, or other amphetamine-like drugs can be given in the morning. Shorter acting immediate release preparations, for example, 5–10 mg, can be used a few times throughout the day as needed, particularly around the circadian nadir at 15:00–17:00. Avoiding these drugs beyond late afternoon is important as they can impair subsequent sleep. These drugs are prescribed carefully given their potential for abuse, psychiatric effects, and cardiovascular consequences [13]. The role for sodium oxybate in EDS requires careful consideration, though the drug is particularly helpful for treatment of severe cataplexy in narcolepsy.

## 6. Safety

Of particular concern is the safety of the individual while driving. Numerous studies have demonstrated that untreated SA is associated with an approximately 2-3 fold increased incidence of traffic accidents [14]. Certainly, insufficient sleep is known to impair safety as well and the loss of a night's sleep has a comparable neuropsychological effect of having a blood alcohol level in the impaired range.

Driving issues should be addressed in the initial consultation. Patients with a history of accidents and untreated sleep disorders represent high risk. Clinicians should ensure they understand their local requirements for reporting sleep disorders to government transportation authorities. While the subjective report of alertness and safety may be reliable for some patients, when there is doubt, obtaining an objective test such as an MWT may be helpful.

## 7. Future Directions

This field is in need of more practical measurement tools for clinical management. New portable devices may be better able to identify impairment in real time. At present, the current neurophysiologic tests are not validated for driving safety. However, if one cannot stay awake at a minimum, the higher order cognition required for driving will certainly be impaired. New medications are being developed given our improved understanding of the mechanisms of sleep-wake control. It is anticipated that drugs will be available in the near future that target the orexin/hypocretin, histamine, and melatonin systems [15].

## Additional Points

Daytime sleepiness is a common presenting concern to physicians. With a careful history, the underlying cause can often be identified. Investigations, including neurophysiological tests, will identify contributing conditions, and a variety of medications are available for management, which can improve quality of life and prevent harm. New tools are under development that will better allow us to quantify sleepiness, and new medications should help facilitate more targeted treatment with fewer side effects.
